# Supplementary material for: Heritability of temperature-mediated flower size plasticity in Arabidopsis thaliana
Source: Quant Plant Biol. 2023 Mar 21;4:e4. doi: 10.1017/qpb.2023.3 (PMC10095859; doi:10.1017/qpb.2023.3)
Supplement: Supplementary file 1 [file qpbsup.zip › S2632882823000036sup001.pdf]

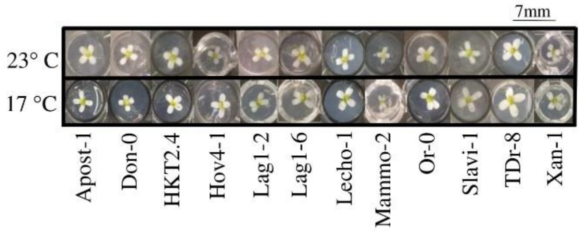

Figure S1. Photos for a representative flower of the 12 accessions grown at 17 °C and 23 °C.

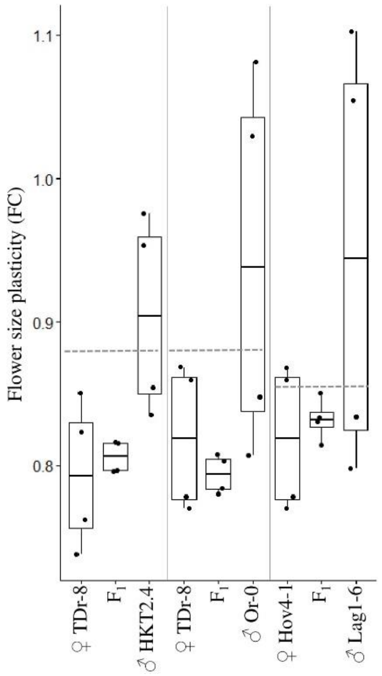

Figure S2. Hybrids showing non-additive inheritance pattern. Hybrids showed significant differences in plasticity to MPV values (shown by dashed line), at a false discovery rate of 10%. p-values were adjusted according to the Benjamini-Hochberg, when n=28).
